# The complete plastid genome sequence of *Neopicrorhiza scrophulariiflora* (Plantaginaceae): an endangered species endemic to The Himalayas regions

**DOI:** 10.1080/23802359.2019.1639560

**Published:** 2019-07-16

**Authors:** Ying-Min Zhang, Zi-Gang Qian, Ai-Li Zhang, Cong-Wei Yang, Guo-Dong Li, Xiao-Li Liu

**Affiliations:** a Faculty of Traditional Chinese Pharmacy, Yunnan University of Chinese Medicine, Yunnan, China;; b Yunnan Key Laboratory for Dai and Yi Medicines, Yunnan University of Chinese Medicine, Yunnan, China

**Keywords:** *Neopicrorhiza scrophulariiflora*, chloroplast genome, medicinal plant, endangered species

## Abstract

*Neopicrorhiza scrophulariiflora* (Pennell) Hong, an endangered perennial species, is endemic to the Eastern Himalayas and Hengduan Mountains. In this study, we have sequenced the complete chloroplast genome of *N. scrophulariiflora*, which is 152,643 bp in length, including two inverted repeat (IR, 25,829 bp) regions, one large single copy region (LSC) and one small single copy region (SSC) of 83,191 bp and 17,794 bp, respectively. The cp genome has 131 annotated genes, including 86 protein-coding genes, 37 tRNA genes, and eight rRNA genes. The overall GC content of it is 38.1%. Phylogenetic analysis using total chloroplast genome DNA sequence of 14 species revealed that *N. scrophulariiflora* was closely relates to two species of *Veronica* with 100% bootstrap value.

*Neopicrorhiza scrophulariiflora* (Pennell) Hong, a perennial herb of the family Plantaginaceae, is endemic to the alpine meadow and scree slope of southwestern China. It distributed in Eastern Himalayas and the Hengduan Mountains ranging from 3600 m to 4200 m in elevation (Li et al. 2016). The long, creeping, and highly bitter rhizomes (Rhizoma Neopicrorhizae) named Huhuanglian are of high medicinal value and have been officially listed in the Pharmacopoeia of the People’s Republic of China for the treatment of fever, jaundice, hemorrhoid, and dysentery (Liu et al. 2011). Because of the impact of global warming and anthropogenic activity, natural populations of this species have suffered rapid declines. It is now categorized as an endangered species in the China Species Red List (Qin et al. 2017). Here, we characterized the complete chloroplast genome sequence of *N. scrophulariiflora* as a resource for conservation and future genetic studies.

Fresh leaves of *N. scrophulariiflora* were collected from Bomi Country (29°46'N, 95°41'E), Tibet Autonomous Region, China and voucher specimens (LiGD20090601) were deposited in Herbarium of Yunnan University of Chinese Medicine. Total genomic DNA was extracted using plant DNA (Bioteke Corporation, China). A library was constructed and sequencing was performed on an Illumina HiSeq 2500 platform (Illumina Inc., SanDiego, CA). A total of 3.1 Gb reads were obtained and de novo assembled using NOVOPlasty (Dierckxsens et al. 2017). The complete cp genome was annotated with the online annotation tool GeSeq (Tillich et al. 2017).

The total chloroplast genome size of *N. scrophulariiflora* is 152,643 bp (GenBank accession No.: MK986819), containing a large single copy (LSC) region of 83,191 bp and a small single copy (SSC) region of 17,794 bp, which are separated by a pair of inverted repeat (IRs) regions of 25,829 bp. The cp genome has 131 annotated genes, including 86 protein-coding genes, 37 tRNA genes, and eight rRNA genes. The overall GC-content of the whole plastome is 38.1%, while the corresponding values of the LSC, SSC, and IR regions are 36.3%, 32.1%, and 43.2%, respectively. A total of 85 SSRs were detected using the online software IMEx (Mudunuri and Nagarajaram 2007). The number of mono-, di-, tri-, tetra-, penta-, and hexa-nucleotides SSRs are 36, 38, 6, 3, 0, and 2, respectively.

To determine the phylogenetic position of *N. scrophulariiflora*, we selected the complete chloroplast genomes of 14 species, including 10 species from Plantaginaceae, and four species from Scrophulariaceae as outgroups. All of the plastomes were aligned using MAFFT v.7 (Katoh and Standley 2013), and the RAxML (Stamatakis 2014) inference was performed using GTR model with support for branches evaluated by 1000 bootstrap replicates (Figure 1). *Neopicrorhiza scrophulariiflora* is found to be closely related to species of the genus *Veronica* compared with species of other genera in Plantaginaceae. The complete chloroplast genome of *N. scrophulariiflora* will provide a useful resource for the conservation genetics of this species as well as for the phylogenetic studies of Plantaginaceae.

**Figure 1.**
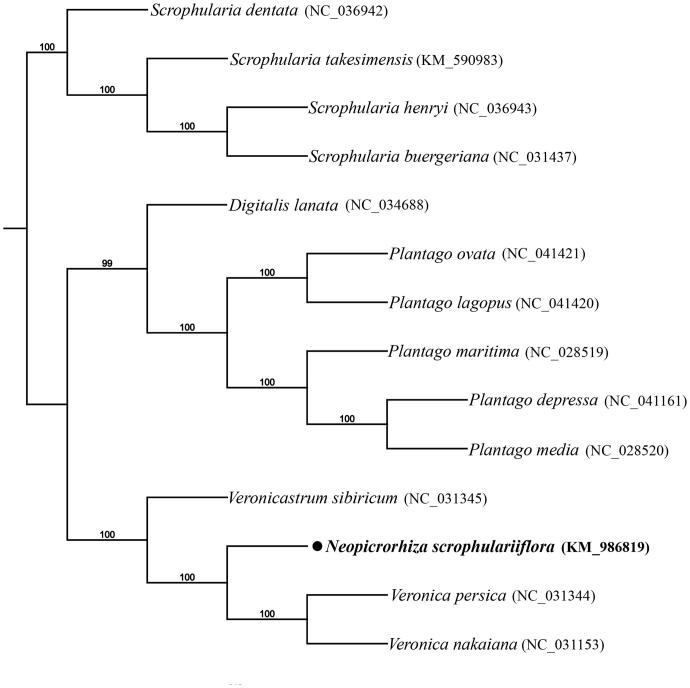
Maximum-likelihood phylogenetic tree inferred from 14 chloroplast genomes. Bootstrap support values >50% are indicated next to the branches.
